# Efficient Preparation of High-Purity Fucoxanthinol by SpyTag-Tailored Active Cholesterol Esterase Aggregates

**DOI:** 10.3390/md20110709

**Published:** 2022-11-12

**Authors:** Wenhui Jin, Ting Yang, Hui Chen, Hua Fang, Weizhu Chen, Quanling Xie, Qian Liu, Yiping Zhang, Zhuan Hong, Guangya Zhang

**Affiliations:** 1 Technical Innovation Center for Utilization of Marine Biological Resources, Third Institute of Oceanography, Ministry of Natural Resources, Xiamen 361005, China; 2 Department of Bioengineering and Biotechnology, Huaqiao University, Xiamen 361021, China

**Keywords:** fucoxanthin, fucoxanthinol, efficient preparation, immobilized enzymes, SpyTag, active aggregates

## Abstract

A novel approach to producing high-purity fucoxanthinol (FXOH) was exploited as a sustainable method to maximize fucoxanthin (FX) utilization. Through fusing the genes of cholesterol esterase and SpyTag and then expressing them in *Escherichia coli*, the fusion chimera was self-assembled into insoluble active aggregates by SpyTag, which could be regarded as carrier-free immobilization. The immobilization yield of the active cholesterol esterase aggregates could reach 60%. They have expressed good activity retention at 92.48% and 60.13% after 3 and 12 cycles, respectively, which is an exciting finding. The conversion ratio of FX to FXOH is 95.02%, which is remarkably higher than those realized via the conventional chemical reduction method (55.86%) and the enzymatic hydrolysis method by free cholesterol esterases (84.51%). The purity of FXOH obtained by this method is as high as 98%, which is much higher than those obtained by other methods. Thus, a promising method for simultaneously purifying and immobilizing active cholesterol esterase aggregates is demonstrated in this study by SpyTag tailoring. In addition, this study provides an eco-friendly method for producing high-purity FXOH from FX in a highly efficient manner.

## 1. Introduction

As a natural carotenoid, fucoxanthin (FX) has important biological activities, including anti-inflammatory, antioxidant, anticancer, hypoglycemic, and lipid-lowering functions, indicating that FX is a natural product with potential medicinal value [1,2,3,4,5,6,7]. Fucoxanthinol (FXOH) is an alcohol derivative and a vital metabolite of FX obtained in vivo from FX through ester bond cleavage and has stronger physiological activity and better bioavailability than FX [8,9,10,11,12]. FXOH is obtained through chemical reduction, enzymatic hydrolysis, and other preparation processes [13,14,15,16]. When FXOH is prepared using the chemical reduction method, the many by-products make its separation and purification difficult, resulting in low yield [15]. The efficient preparation of FXOH from FX remains a challenge due to the coexistence of multiple by-products with similar polarity in the chemical reduction system.

Therefore, the enzymatic hydrolysis method is more suitable for preparing FXOH than the chemical reduction method. Many hydrolases, including lipases and esterases, are available. Nevertheless, the relatively high cost of lipases and esterases coupled with low operational stability remains a major techno-economic bottleneck that limits their widespread industrial applications [17,18,19]. In recent years, purification techniques in the laboratory have often used purification tags to purify the enzymes, including the histidine tag, a common tag based on affinity, to purify enzymes by using a nickel column. These methods can make the purity of the target enzyme reach 90% or higher [20]. However, His-tag-based chromatography is expensive, time-consuming, and difficult to scale-up [21]. Thus, research and development of low-cost immobilized enzymes are vital to solve this problem. In particular, protein engineering offers the potential to shift enzyme properties and offers the discovery of entirely new reactions, so biocatalysis can be translated from laboratory to industrial process [19].

The immobilization of enzymes means that a free and soluble enzyme is transformed into an insoluble enzyme that retains catalytic activity after being treated with specific methods [22,23]. Nowadays, the improvement of many enzyme features is a potent tool. Immobilization can be coupled with enzyme purification via diverse strategies, such as immobilization on pre-existing porous supports, multipoint covalent attachment on support surfaces, and physical or chemical modification with polymers. Carrier-free immobilization is another strategy for preparing heterogeneous biocatalysts, such as cross-linked enzyme aggregates (CLEAs), cross-linked enzyme crystals (CLECs), and bio-orthogonally cross-linked enzymes (PR-CLEs). If they are properly designed, immobilization can also positively alter enzyme activity, selectivity, specificity, and inhibitions. Immobilized enzyme stability causes may be ascribed to the prevention of intermolecular processes, the chemical modification of soluble enzymes, or molecular biology via directed evolution and site-directed mutagenesis, but not all immobilization processes may result in enzyme stabilization, and an improperly designed immobilized enzyme biocatalyst may have a far worse performance than the soluble enzyme, including lower activity and stability [24,25,26,27]. Compared to other carrier-free immobilization methods, active aggregates do not need additional cross-linking agents and can combine the expression and immobilization of recombinant enzymes in *Escherichia coli*, which is conveniently prepared in the pure form. Although the immobilized enzyme in this way has low mechanical strength, integrating enzyme purification and immobilization processes into one step can significantly simplify the preparation process, and reduce the total preparation cost [28,29,30,31]. SpyTag and SpyCatcher originate from the autocatalytic isopeptide bond-forming subunit of *Streptococcus pyogenes’* fibronectin-binding protein. They undergo spontaneous intermolecular isopeptide bond formation when mixed. The reaction can proceed rapidly in mild conditions, and the bond is robust to diverse harsh conditions [21,32,33]. Moreover, due to the formation of active aggregates caused by the addition of new short peptide tags, the target enzyme can be quickly separated from impurities by simple centrifugation and has enzymatic activity. Compared with induced inclusion bodies, their denaturation and renaturation are unnecessary, and direct use can significantly reduce the cost of the purified enzyme [34,35,36,37,38]. This tailored enzyme also has the advantage of low production cost because active aggregates can be reused in the production process to reduce environmental pollution and production costs [39,40,41].

Although the biological activity of FXOH has been widely investigated in vitro and in vivo [42,43,44,45], the efficient preparation of FXOH applicable to industrial production has not been reported. This study proposes a green and efficient method for preparing high-purity FXOH from FX by SpyTag-tailored active cholesterol esterase aggregates, a monomer enzyme as a hydrolase. This method has many advantages, such as a high-purity product, environment-friendly technology, and the ability to be reused regularly. This discovery contributes to the maximum utilization of active cholesterol esterase aggregates and further exploits FXOH.

## 2. Results and Discussion

### 2.1. Preparation of FXOH by Chemical Reduction

**Figure 1:**
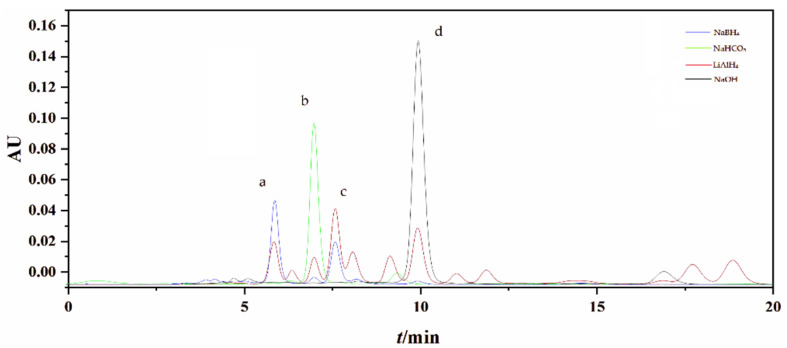
Liquid chromatogram of FX chemical reduction reaction by using (**a**) NaBH_4_ (blue), (**b**) NaHCO_3_ (green), (**c**) LiAlH_4_ (red), and (**d**) NaOH (black). (FX: *t*_R_ = 7.22 min; FXOH: *t*_R_ = 6.18 min).

FXOH was obtained by adding an appropriate amount of a reducing agent to an ethanol solution with 50% FX as the initial raw material. As shown in Figure 1, the comparison of reaction conditions under different reducing agents, such as sodium borohydride, lithium aluminum hydride, sodium hydroxide, and sodium bicarbonate, indicated that FXOH was detected in the reduction products when sodium borohydride and lithium aluminum hydride were used as reducing agents. No FXOH was found in the reduction products of the two other reducing agents. When lithium aluminum hydride was used as the reducing agent to prepare FXOH, as many as 11 new compounds were observed. Consequently, the subsequent separation and purification were difficult because of the large number of by-products. Among the reducing agents, sodium borohydride had fewer by-products and a higher relative yield of FXOH and was preferred as a reducing agent for FXOH preparation via the chemical reduction method.

By optimizing chemical reduction conditions, we found that the relative content of FXOH increased with the amount of sodium borohydride, reaction time, and reaction temperature. When the dosage of sodium borohydride as the reducing agent reached 5%, the relative content of FXOH increased slowly and reached the maximum when the reducing dosage was 10%. The relative range of FXOH no longer increased as the dosage of the reductant increased but showed a downward trend. The addition of a reducing agent might react with the resulting FXOH converted into other substances. Therefore, a reducing agent dose of 10% was the best condition for the reaction. Similarly, when the reaction time was 3 h, the relative content of FXOH was highest. With a prolonged reaction time, the relative content of FXOH did not increase but showed a downward trend. The prolonged reaction time might have converted the generated FXOH into other substances again. Accordingly, the reaction time of 3 h was chosen. When the reaction temperature was 37 °C, the relative content of FXOH was highest. Nevertheless, with increased reaction temperature, the relative content of FXOH decreased, which might be due to the unstable change in FXOH stored at a high temperature for a long time. According to the properties of reactants, the reaction temperature of 37 °C was determined to be the most suitable. In conclusion, according to the optimization results, the optimal reaction conditions for the preparation of FXOH by chemical reduction were as follows: reducing agent, sodium borohydride; reducing dosage, 10%; reaction time, 3 h; and reaction temperature, 37 °C.

The crude product of FXOH was prepared through the above method, separated using a reversed-phase chromatographic column under reduced pressure, and eluted with a 50:50–100:0 methanol solvent system for gradient elution. Eluents were collected and concentrated under reduced pressure to obtain FXOH. The purity and yield of FXOH were 85.51% and 55.86%, respectively.

Preparing FX derivatives via the chemical reduction method was important for enriching the number of FX derivatives and further studying their activity. However, the practice of FXOH brought difficulties to the subsequent separation and purification with increased losses and low yield.

### 2.2. Enzymatic Preparation of FXOH

**Figure 2:**
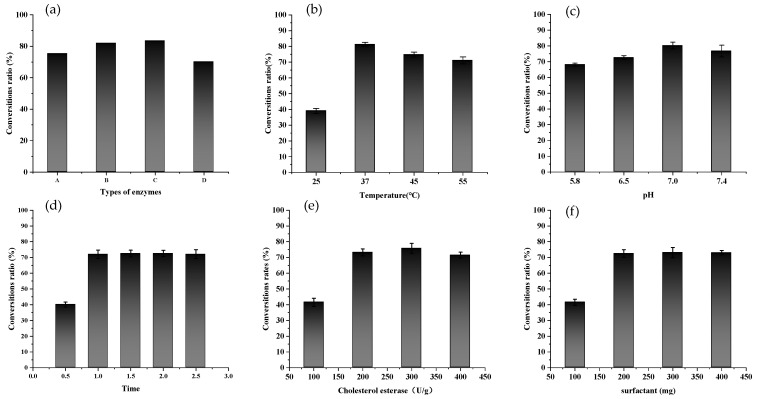
Effect of reaction conditions on the conversion ratio of FX to FXOH: (**a**) enzyme type (A: porcine pancreatic lipase; B: lipase (Brand: Klamar); C: cholesterol esterase; D: *Candida rugosa* lipase), (**b**) enzymatic temperature, (**c**) enzymatic pH, (**d**) enzymatic time, (**e**) cholesterol esterase dose, and (**f**) surfactant dose.

The same substrate concentration of FX was used for enzymatic preparation as for chemical reduction. Figure 2a shows that all four enzymes could promote the conversion of FX into FXOH. When lipases, especially animal-derived lipase, are used as hydrolases, they can obtain FXOH by hydrolysis of FX with a sufficiently high yield [14]. However, esterases display broad substrate specificity, catalyzing the hydrolysis of not only ester bonds of short-chain fatty acids ester but also non-ester bonds, such as thiols, amides, and carbamates. There is a prominent interest lately in utilizing microbes for the large-scale production of lipases and esterases, which is in line with the high demand for various industrial applications of microbial esterases [17].

After 1.5 h reaction, the conversion ratio of FX to FXOH was highest, reaching 83.62%. Therefore, cholesterol esterase was selected as the most suitable enzyme to continue the optimization of enzymatic hydrolysis conditions. As shown in Figure 2b, the conversion ratio of FX to FXOH varied with enzymatic temperature. The highest and lowest conversion ratios were observed at 37 °C and 25 °C, respectively, which was related to the optimum temperature of the enzyme. FXOH was sensitive to heat and increased with increasing temperature. FXOH was unstable, resulting in a decreased conversion ratio. Hence, the optimum enzymatic temperature for cholesterol esterase was 37 °C.

At the same time, phosphate buffer with pH between 5.8 and 7.4 was placed in the reaction system, and the reaction was carried out at 37 °C for 1.5 h to investigate the effect of system pH on the conversion ratio of FX to FXOH. From Figure 2c, the conversion ratio of FX to FXOH was meager under low-pH conditions and was inconsiderably different from those under neutral and alkaline conditions. The optimum enzymatic pH was 7.0, which was also consistent with the optimum pH of the available enzymes.

According to Figure 2d, the conversion ratio of FX to FXOH increased with prolonged enzymatic reaction. However, within the first hour of reaction, the conversion ratio of FX to FXOH rose rapidly, but after 1 h, almost no change was observed, and the response was relatively stable. In Figure 2e, the conversion ratio of FX to FXOH also increased with increases in the amount of cholesterol esterase. The highest conversion ratio reached 75.81% when the amount of cholesterol esterase was 300 U/g. Afterward, the conversion ratio of FX to FXOH did not increase even if the amount of cholesterol esterase increased again. As shown in Figure 2f, sodium taurocholate was similar to cholesterol esterase. When the surfactant amount reached 200 mg, the conversion ratio of FX to FXOH was highest (up to 73.14%). Then, the conversion ratio of FX to FXOH did not rise when the surfactant amount was increased.

In summary, the optimal conditions for the preparation of FXOH by enzymatic hydrolysis were as follows: FX substrate, 20 mg; amount of cholesterol esterase, 300 U/g; amount of sodium taurocholate surfactant, 200 mg; pH of the enzymatic system, 7; reaction medium, water; reaction temperature, 37 °C; and reaction time, 1.5 h. The average conversion ratio of FX to FXOH prepared by enzymatic hydrolysis was 84.51%, the standard deviation was 3.99%, and the purity of FXOH was 90.79%.

### 2.3. Preparation of Active Cholesterol Esterase Aggregates by SpyTag Tailoring

**Figure 3:**
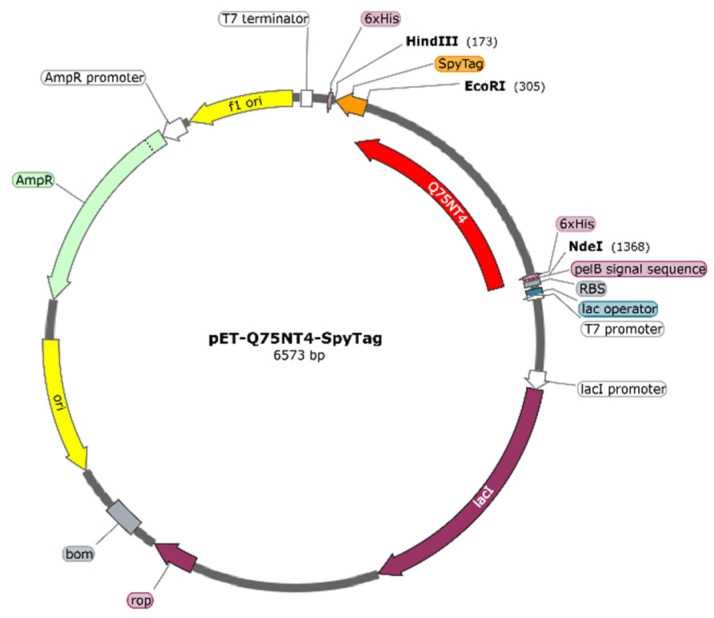
Profiles of plasmid Pet-Q75NT4-SpyTag.

**Figure 4:**
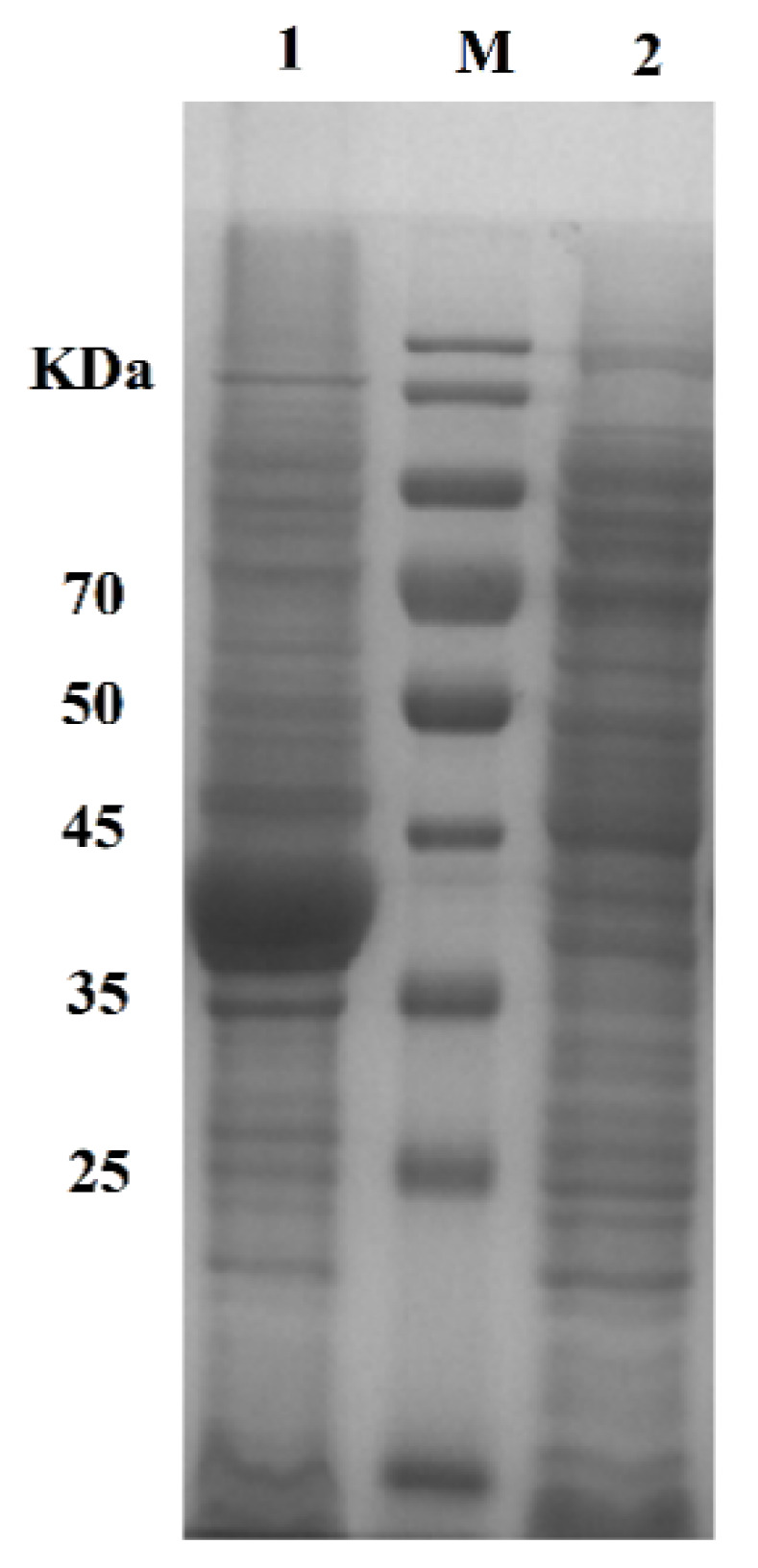
SDS-PAGE results of Q75NT4 protein. M: marker, lane 1: precipitate of Q75NT4 recombinant bacteria after crushing and centrifugation, lane 2: supernatant of Q75NT4 recombinant bacteria after crushing and centrifugation.

We designed cholesterol esterase (Q75NT4) containing SpyTag. The fusion gene Pet-Q75NT4-SpyTag was transformed and induced, and SpyTag was terminally fused to Q75NT4. Figure 3 shows the diagrammatic sketch of the Pet-Q75NT4-SpyTag chimera design. Pet-Q75NT4-SpyTag was overexpressed and induced by isopropyl β-d-1-thiogalactopyranoside (IPTG), as shown by sodium dodecyl sulfate–polyacrylamide gel electrophoresis (SDS-PAGE) (Figure 4). The results showed that the Pet-Q75NT4-SpyTag protein was successfully overexpressed in the precipitates (Figure 4, lane 1) and weakly expressed in supernatants (Figure 4, lane 2), which were purified using a simple centrifugation method. For the purity of Pet-Q75NT4-SpyTag, SDS-PAGE yielded one band between 35 and 45 kDa (Figure 4, lane 1), which matched the theoretical molecular weight value at 42.9 kDa calculated by the ProtParam.

The absorbance values of the precipitate and supernatant at 500 nm were measured using the UV–vis method to determine the enzyme activity. According to the results of the enzyme activity, the precipitate could catalyze the hydrolysis of cholesterol oleate ester and had cholesterol esterase activity, indicating that the target enzyme Q75NT4 was expressed in the form of active cholesterol esterase aggregates in *Escherichia coli*.

**Figure 5:**
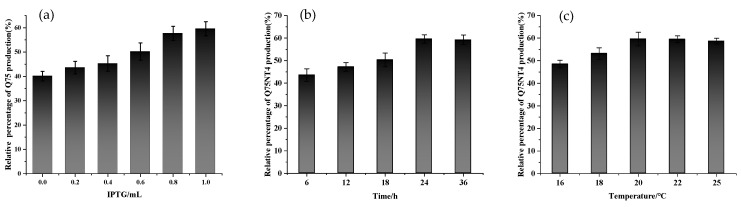
Yield of active cholesterol esterase aggregates under different conditions: (**a**) amounts of IPTG, (**b**) induction times, and (**c**) induction temperatures.

Induction conditions were optimized on the basis of the yield of active cholesterol esterase aggregates. As shown in Figure 5, when the IPTG addition amount was 1 mL, the induction temperature was 20 °C. The induction time was 24 h, and optimal induction conditions were obtained. The average yield of active cholesterol esterase aggregates was 59.79%, based on 1 L medium (Table 1).

Two products, i.e., inactive inclusion body and active aggregates, may be formed in the thallus by introducing a short peptide tag to bind to the target protein. For the inclusion body, denaturation and renaturation are required to recover the enzyme activity, but the operation is cumbersome. The formation of such active aggregates is important in the expression and purification of enzymes. The tag introduced in this paper is SpyTag, a short amphiphilic peptide with a sequence of hydrophilic and hydrophobic residues. Binding to cholesterol esterase may promote the assembly of the target protein into nano-sized protein aggregates, which may be due to the introduction of the amphiphilic tag. The intermolecular diffusion and changes in some specific intermolecular forces result in the formation of protein aggregates with stable structures [46].

Active protein aggregates, commonly referred to as inclusion bodies, are found in *Escherichia coli* and obtained from overexpression [29]. Various biotechnological applications are possible with these active protein aggregations induced by peptides and formed by the spontaneous expression of fusion genes in vivo. We introduce a novel in vitro controllable active enzyme particle. However, for the first time, we found that cholesterol esterase became insoluble active aggregates when induced by SpyTag. Initially, these particles exhibited a narrow distribution. After purification, they displayed a micro size and visible polydispersity. This result was comparable with immobilized cholesterol esterase on microcarriers formed by SpyTag’s self-assembly.

The active aggregates represent a promising strategy for immobilizing enzymes without additional carriers and chemicals. Compared with other carrier-free immobilization methods, active aggregates do not need additional cross-linking agents and can combine the expression, purification, and immobilization of target enzymes. Nevertheless, due to the nature of the assembly and aggregation of proteins, there are still problems of structural instability, such as low catalytic activity and soft matrix [47].

### 2.4. Morphological Analysis of Active Cholesterol Esterase Aggregates

**Figure 6:**
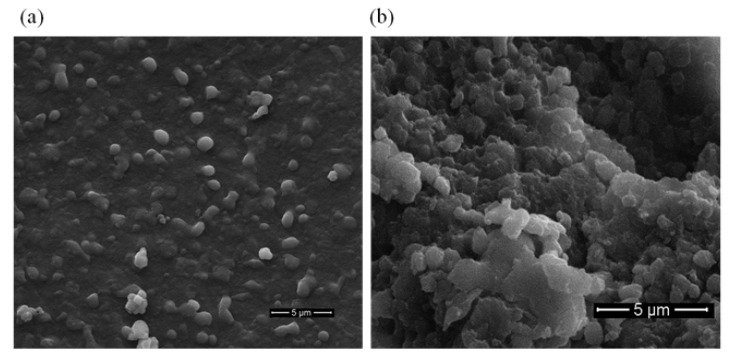
SEM images of active cholesterol esterase aggregates: (**a**) 5000× and (**b**) 10,000× (after mill).

Scanning electron microscopy (SEM) was used to analyze active cholesterol esterase aggregates. SpyTag-induced nanospheres had diameters between 500 and 800 nm (Figure 6), and this finding was consistent with those in previous reports [34].

Figure 6 depicts the SEM images of active cholesterol esterase aggregates before and after mill. Figure 6a shows that the microstructure of cholesterol esterase aggregates induced by SpyTag before mill was round and granular. Fused nanospheres had diameters of 500–800 nm, and this finding was similar to that observed in Figure 6b after mill. This phenomenon might be due to the aggregation of many crude proteins and cholesterol esterase proteins during the formation of crude active cholesterol esterase aggregate particles or the accumulation of cholesterol esterase itself to form a bulk particle structure.

### 2.5. Kinetic Parameters of Active Cholesterol Esterase Aggregates

To illustrate the change in free cholesterol esterase kinetic parameters and active cholesterol esterase aggregates, we calculated K_m_ from the Michaelis–Menten equation. The results are shown in Table 2. The K_m_ and V_max_ values of free and insoluble active cholesterol esterase aggregates were 4.88 × 10^−2^ and 6.25 × 10^−2^ mmol/L, respectively. As mentioned previously, the average size of the active cholesteryl esterase aggregates was about 500–800 nm, which hindered the diffusion of enzyme and substrate. Therefore, the K_m_ of the active cholesterol esterase aggregates was higher than that of the free cholesterol esterase.

In addition, the catalytic efficiency (k_cat_/K_m_) of the free cholesterol esterase was 27.87 mmol·s/L, and the k_cat_/K_m_ of the active aggregates of cholesterol esterase was 17.92 mmol·s/L. These results might be due to the fact that the active aggregates reduced the active site of cholesterol esterase and increased the mass transfer barrier because of the steric hindrance of the fetoprotein, resulting in decreased catalytic efficiency. Compared with that of the free enzyme, the enzymatic activity of the induced active aggregates might be increased, decreased, or unchanged, which was consistent with the results of most studies.

### 2.6. Reusability of Active Cholesterol Esterase Aggregates

**Figure 7:**
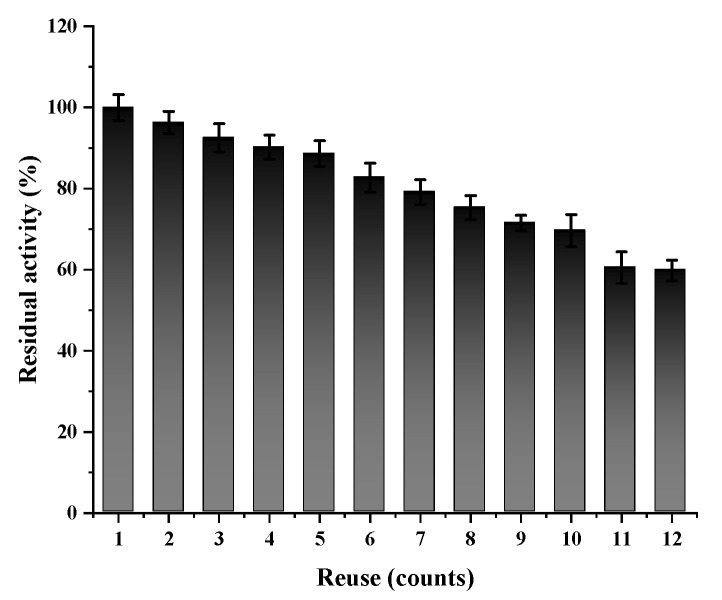
Reuse of active cholesterol esterase aggregates.

Only the active aggregates of immobilized enzymes can be separated from the substrate and other impurities for reuse by simple centrifugation. After simple centrifugation, the active aggregate, including the immobilized enzyme, could be separated from the substrate and other impurities to reuse. The reusability of the active cholesterol esterase aggregates was assayed to evaluate their quality and industrial potential. This study recycled the active cholesterol esterase aggregates for 12 cycles. According to Figure 7, the active cholesterol esterase aggregate activity declined slowly with increased enzyme usage. The active cholesterol esterase aggregates had good retention of 92.48% and 60.13% after 3 and 12 cycles, respectively. The proposed method was well-operational compared with other conventional methods.

The results showed that the reusability of active cholesterol esterase aggregates was good and that the purpose of reuse could be achieved by simple centrifugation in the later stage. This method is convenient, economical, and feasible.

### 2.7. Preparation and Purification of FXOH by Using Different Methods

At the same initial substrate (FX) concentration, the transformation of FXOH products, including chemically reduced, free, and active cholesterol esterase aggregates, under different preparation methods, was qualitatively analyzed by high-performance liquid chromatography. Compared with the chemical reduction method that generated multiple by-products, the enzymatic preparation methods for FXOH saved considerable costs in separation and purification and could obtain a higher conversion ratio of FX to FXOH and high purity of FXOH. As shown in Table 3, the conversion ratio of FX to FXOH by using active cholesterol esterase aggregates was 95.02%, which was remarkably higher than those realized via the conventional chemical reduction method (55.86%) and the enzymatic hydrolysis method with free enzyme (84.51%); however, the cost of this method was the lowest. Miyashita provided a four-step process based on porcine-pancreas-derived lipase for the production of FXOH from FX. The ratio of enzyme to substrate was 20:1. Although the conversion ratio of this process was 82.8%, it used a large number of enzymes for the substrate conversion, which could have an impact on the economic feasibility of the process [14].

Therefore, the cost of the enzymes means that, in many cases, they must be reused multiple times to make the process economically viable. Accordingly, reuse of the enzyme is essential; otherwise, a continuous operation is required. In either case, immobilization is a crucial tool to separate or retain enzymes. Given that the economy is vital to the viability of the final process, even minor improvements in observed activity can have a marked benefit to the process.

In this study, active cholesterol esterase aggregates could be efficiently and repeatedly used to generate FXOH, dramatically reducing production costs. The purity of FXOH is above 98% in this method; it can prepare high-purity FXOH in circulation, providing technical support for its development and application.

## 3. Materials and Methods

### 3.1. Chemicals and Reagents

FX was extracted and refined from brown algae, as described previously [48], and prepared and identified in our laboratory (purity ≥ 50%) [49,50]. Analytically pure sodium borohydride, lithium aluminum hydride, sodium hydroxide, and sodium bicarbonate were obtained from National Pharmaceutical Holding Chemical Reagent Co., Ltd. (Shanghai, China). All enzymes, such as porcine pancreatic lipase, *Pseudomonas* cholesterol esterase, and *Candida rugosa* lipase, were obtained from Sigma-Aldrich (St Louis, MO, USA). Lipase (Brand: Kramer) was obtained from Ziyi Reagent Factory (Shanghai, China). The protein ladder was acquired from Thermo Fisher (Waltham, MA, USA). *Escherichia coli* strain BL21 (DE3) was preserved in our laboratory. HPLC-grade methanol was obtained from Merck KGaA (Darmstadt, Germany). Ultrapure water was produced using a Millipore Milli-Q system (Millipore Corp., Billerica, MA, USA). The chemical reagents used in this research were of analytical grade and purchased from Sigma-Aldrich (Saint Louis, MO, USA).

### 3.2. Preparation of FXOH by Chemical Reduction

A round bottle flask was filled with crude FX, reducing agent, absolute ethyl alcohol, and magnetic stirrer. The flask was then heated in a magnetic heating device with an oil bath equipped with a condensing tube to the set temperature and maintained under condensation reflux for 1–3 h. The reaction parameters were optimized by screening the types of reducing agents (sodium borohydride, lithium aluminum hydride, sodium hydroxide, and sodium bicarbonate) and changing the reaction temperature (25 °C, 37 °C, 50 °C, and 60 °C), reaction time (0, 1, 2, and 3 h), and the ratio of the reducing agent (1%, 5%, 10%, and 20%; *w*/*w*).

The reaction situation was detected by high-performance liquid chromatography. The mixed derivative solution was combined and evaporated in a vacuum to yield a crude extract. The natural extract was subjected to ODS C18 column (30 mm × 400 mm, methanol–H_2_O, 50:50–100:0) to generate separated and purified fractions. The FXOH was obtained from the experiment.

### 3.3. Preparation of FXOH by Enzymatic Hydrolysis

Crude FX, enzymes, and sodium taurocholate were added to a conical flask in proportion as surfactants. Phosphate buffer was added to prepare FXOH by enzymatic hydrolysis. The effect of enzymatic hydrolysis factors on the conversion ratio of the reaction was determined by single-factor experiments, including screening of the enzyme type (i.e., porcine pancreatic lipase, cholesterol esterase, lipase (Brand: Kramer), and Candida rugosa lipase), enzymatic pH (i.e., 5.8, 6.5, 7.0, and 7.4), enzymatic time (0.5, 1, 1.5, 2, and 2.5 h), enzymatic temperature (25 °C, 37 °C, 45 °C, and 55 °C), enzyme dose (1:5, 1:10, 1:15, and 1:20; *w*/*w*), and catalyst dose (1:5, 1:10, 1:15, and 1:20; *w*/*w*). The conversion ratio of FX to FXOH was used as readout for results. Each experiment was performed in triplicate. The conversion ratio of FX to FXOH was determined as follows:

(1) $$\mathrm{FXOH}\;\mathrm{conversion}\;\mathrm{ratio}\;(\%)\;=\frac{m_{\mathrm{FXOH}}\times M_{\mathrm{FXOH}}}{m_{\mathrm{FX}}\times M_{\mathrm{FX}}}\times 100$$

where m_FXOH_ denotes the amount of FXOH obtained from the reaction (mg); M_FXOH_ and M_FX_ refer to the molecular weight of FXOH (617) and FX (659), respectively; and m_FX_ denotes the mass of FX as a substrate.

### 3.4. Protein Design and Gene Construction

The Q75NT4 gene (*Pseudomonas* origin) (https://www.uniprot.org/uniprot/Q75NT4, updated on 5 July 2004) was designed, and its signal peptide was removed. Genes were designed, synthesized, and verified by the Suzhou GENEWIZ Company (suzhou, China). The combining linker (AEAAAKEAAAKEAAAKA) was used with the SpyTag lichenase (Gene ID:937470), and Q75NT4 gene sequences were sequentially cloned into Pet-22b(+). The plasmid Pet-Q75NT4-SpyTag (Figure 1a) was transformed into *Escherichia. coli* strain BL21 (DE3).

### 3.5. Protein Expression and Purification

The plasmid was cotransformed into *Escherichia coli* BL21 (DE3), which was cultured for 12 h in a Luria–Bertani medium containing ampicillin (100 μg/mL) at 37 °C and shaken at 200 rpm in a shaking incubator (Tensuc, Shanghai, China). Afterward, expression was initiated on terrific broth from the starter culture.

Once the optical density (OD_600_) reached 0.6 after 3–4 h, IPTG was added to the culture, bringing the final concentration to 0.5 mmol/L. The culture was then incubated at 20 °C for 24 h. After the end of the culture, *Escherichia coli* cells were collected by centrifugation at 12,000× *g* for 20 min at 4 °C. The cells were resuspended with cold PBS (pH 7.0) and lysed by ultrasonication on ice [21]. The supernatant and precipitate were collected separately by centrifugation at 4 °C (12,000× *g*, 10 min). The insoluble fractions were active cholesterol esterase aggregates. The aggregates were washed once with PBS and then resuspended with the same volume of PBS [34].

The amounts of active cholesterol esterase aggregates in both fractions were determined densitometrically through denaturing polyacrylamide gel electrophoresis by using 12% precast gels. The protein concentration was determined using BSA as the protein standard via the Bradford method. With the data above collected, giving some critical parameters that define the immobilization process becomes possible [51].

### 3.6. Enzyme Activity Determination

The activity of cholesterol esterase was determined using the continuous spectrophotometric rate determination method. The procedure used was that described at [52]. The steps are as follows: The reaction system consisted of 2.1 mL of potassium phosphate buffer (400 mM), 0.05 mL of 15% (*w*/*v*) taurocholic acid solution, 0.05 mL of 15% (*w*/*v*) cholic acid solution (Chol), 0.1 mL of peroxidase enzyme solution, 0.5 mL of 8.6 mM cholesteryl oleate solution (Chol-Oleate), and 0.05 mL of 5% (*w*/*v*) phenol solution mixed by inversion and equilibrated to 37 °C. The mixture was added to 0.05 mL of 1.76% (*w*/*v*) 4-amino antipyrine solution and 0.05 mL of cholesterol oxidase enzyme solution (Chol Oxid) mixed by inversion, and the baseline absorbance was obtained at 500 nm. After approximately 5 min, the mixture was added to 0.05 mL of cholesterol esterase solution and immediately mixed by inversion. The increase in A_500nm_ was recorded for approximately 10 min. The ΔA_500nm_ /min was obtained using the maximum linear rate for test and blank samples. One unit hydrolyzed 1.0 µmol cholesteryl oleate to cholesterol and oleic acid per minute at pH 7.0 at 37 °C in the presence of taurocholate.

### 3.7. Morphological Analysis of Active Cholesterol Esterase Aggregates

Active cholesterol esterase aggregates were washed with deionized water for SEM, completely dried overnight in a drying oven maintained at 60 °C, and characterized through gold sputtering by using an S-4800 scanning electron microscope (Hitachi, Japan) equipped with an energy-dispersive X-ray spectroscope.

### 3.8. Determination of Kinetic Parameters for Cholesterol Esterase

The kinetic parameters for cholesterol esterase were obtained using the following substrate concentrations: 0.1, 0.2, 0.5, 1.0, 1.5, and 2.0 mol/L. K_m_ and V_max_ values were calculated using the double-reciprocal method. The K_m_ and V_max_ values of the free cholesterol esterase and active cholesterol esterase aggregates were calculated using the Lineweaver–Burk plot method. All enzymatic assays were performed in triplicate.

### 3.9. Reusability of Active Cholesterol Esterase Aggregates

After each cycle, reusability was determined by measuring the activity of the active cholesterol esterase aggregates. After the measurement, the active cholesterol esterase aggregates were removed from the reaction system by centrifugation (12,000× *g*, 1 min) at 4 °C and thoroughly washed with 0.2 mol/L PBS. Then, a new substrate solution was added to the reaction system containing active cholesterol esterase aggregates to start a new enzymatic reaction cycle. Twelve rounds were performed. The specific activity of the first round was defined as 100%. All experiments were carried out in triplicate.

### 3.10. Analysis of FXOH by Liquid Chromatography

Analyte separations were performed on a Waters 2695 system equipped with a diode array detector (Waters, Milford, MA, USA) by using a SB C18 column (4.6 mm × 250 mm, 5 μm; Agilent Technologies, Inc., Santa Clara, CA, USA) maintained at room temperature. The mobile phase was composed of methanol and water (92:8, *v*/*v*) at a flow rate of 1.0 mL/min, and the injection volume was 10 µL [8].

## 4. Conclusions

This experiment studied the purity and conversion ratio of FX to FXOH prepared using different methods and developed a novel enzymatic routine for the efficient preparation of high-purity FXOH. Low-cost active cholesterol esterase aggregates obtained by SpyTag tailoring were used as a new type of immobilized enzyme to prepare FXOH. This method integrated separation and purification into one step. The active cholesterol esterase aggregates exhibited good enzymatic activity, especially their efficient reusability, thereby promoting convenient preparation and potential industrial applications. The results of this study also provided a new idea for preparing carrier materials for protein purification and immobilization.

## Figures and Tables

**Table 1:** Yield of active cholesterol esterase aggregates.

Enzyme	Concentration of Bacteria a/(g/L)	Concentration of Precipitates a/(g/L)	Mass of Active Aggregates b/(g/g)	Average Yield (%)
Q75NT4	14.10 ± 0.92	7.99 ± 0.63	0.57 ± 0.01	60 ± 3
	15.81 ± 1.26	9.62 ± 0.72	0.61 ± 0.02	
	15.29 ± 1.19	9.45 ± 0.69	0.62 ± 0.02	

Note: a: measured by the mass of assay in 1 L medium, b: measured by the mass of assay in 1 g bacterial cell.

**Table 2:** Kinetic parameters of free cholesterol esterase or active cholesterol esterase aggregates.

Enzyme	K_m_ (mmol/L)	k_cat_/(s^−1^)	k_cat_/K_m_ (mmol/L/s)
Free Cholesterol Esterase	4.88 × 10^−2^ ± 0.01	1.36 ± 0.29	27.87 ± 2.37
Active Cholesterol Esterase Aggregates	6.25 × 10^−2^ ± 0.02	1.12 ± 0.18	17.92 ± 2.06

**Table 3:** Comparison of parameters and costs by three preparation methods.

Preparation Method	Product Type	Conversion Ratio (%)	Cost (USD/mg)
Chemical reduction	FXOH, multiple by-products	55.86	2500.00
Free enzyme	FXOH	84.51	2000.00
Immobilized enzyme	FXOH	95.02	300.00

Note: The purity of the final product FXOH is >98%.

## Data Availability

All data supporting the conclusions of this article are included in this article.
